# Argatroban for intraoperative anticoagulation in a patient with history of heparin-induced thrombocytopenia and end-stage renal disease undergoing left atrial appendage occlusion: a case report

**DOI:** 10.3389/fmed.2025.1690340

**Published:** 2025-11-05

**Authors:** Kevin Eappen, Kendra L. Walsh, Beverly Ejiofor, Andrew Maslow, Shyamal Asher

**Affiliations:** ^1^The Warren Alpert Medical School of Brown University, Providence, RI, United States; ^2^Department of Anesthesia, Critical Care, and Pain Medicine, Massachusetts General Hospital, Boston, MA, United States; ^3^Department of Anesthesiology, Brown University Health, Providence, RI, United States

**Keywords:** heparin-induced thrombocytopenia, HIT, LAAO, renal impairment, argatroban

## Abstract

Heparin is the standard anticoagulant for structural cardiac procedures, including left atrial appendage occlusion (LAAO). However, alternative agents are needed in patients with contraindications such as heparin-induced thrombocytopenia (HIT). Data on the use of argatroban, a direct thrombin inhibitor, for procedural anticoagulation during LAAO are extremely limited. We describe a 67-year-old man with chronic atrial fibrillation, end-stage renal disease on hemodialysis, and a history of HIT type II who underwent LAAO with a Watchman device under general anesthesia. Due to his renal failure and high risk of recurrent HIT, argatroban was selected for intraoperative anticoagulation. A reduced initial bolus of argatroban achieved supratherapeutic activated clotting time (ACT), and when the infusion was started, ACT levels again exceeded the target range, highlighting the need for close monitoring. The procedure was completed without thromboembolic or hemorrhagic complications. This case demonstrates the effective use of argatroban as an intraoperative anticoagulant in LAAO for patients with HIT and renal impairment. A lower initial bolus and infusion rate may be sufficient with vigilant ACT monitoring to avoid complications of prolonged anticoagulation.

## Introduction

Anticoagulation is critical in structural heart interventions to reduce the risk of intraoperative thromboembolism (1). In patients with atrial fibrillation, daily anticoagulation decreases the risk of thrombus formation in the left atrial appendage, which could lead to cerebrovascular events (2). Left atrial appendage occlusion (LAAO) is a transcatheter procedure indicated in patients with chronic atrial fibrillation who cannot tolerate anticoagulant pharmacotherapy. Heparin is the standard intraoperative anticoagulant in LAAO as well as other transcatheter cardiac procedures (1) and is rapidly reversible with protamine. However, if heparin is contraindicated, such as in patients with a confirmed history of heparin-induced thrombocytopenia (HIT), a safe and reliable alternative anticoagulant must be administered (3).

Argatroban is a direct thrombin inhibitor that is primarily metabolized and eliminated through the hepatobiliary system; it must be dose-reduced in Child-Turcotte-Pugh classes B and C liver disease but can be used with any degree of renal impairment (4, 5). This agent is commonly used to treat patients with active HIT (6) and can be used for procedural anticoagulation when heparin is contraindicated (Table 1). Alternatively, bivalirudin, a derivative of hirudin, the anticoagulant peptide in leech saliva, is another option for patients who cannot receive heparin. Similar to argatroban, bivalirudin directly inhibits the activity of thrombin, but due to its significant renal clearance, it requires dosage adjustments in the setting of kidney disease. As a demonstration of this point, in healthy adults, the half-life of bivalirudin is approximately 25 min; this increases to approximately 3.5 h in patients receiving intermittent hemodialysis (7, 8).

**Table 1:** Selected literature review of intraoperative argatroban anticoagulation in cardiac procedures from the PubMed search since 2010.

Procedure	Patient background	ACT goal (seconds)	Argatroban dose	ACT response	Complications	Source
Transcatheter ablation of atrial fibrillation or ventricular arrhythmia	14 patients, contraindications to heparin	300–350	Bolus: 350 mcg/kg Then infusion: 25 mcg/kg/min	Not specified	None reported	Voskoboinik et al. 2020 (28)
Radiofrequency catheter ablation of atrial fibrillation	81-year-old man, paroxysmal AF, heparin resistance (type-1 antithrombin III deficiency)	300–400	After 20,000 units of heparin: Bolus: 10 mg Infusion: 1.6 mg/kg/min	391 initial, maintained 300–400 during case	None	Kang et al. 2019 (29)
Radiofrequency catheter ablation of atrial fibrillation	74-year-old man with CAD, CHF, DM, HTN, sleep apnea, CKD, and prior HIT	300–400	Bolus: 3 mg, then an additional 7 mg bolus, continuous infusion at 3 mg/h (0.7 μg/kg/min), another 3 mg bolus	ACT reached 381 s, maintained at 300–400 s with 15-min monitoring	None	Sakai et al. 2022 (30)
Transcatheter aortic valve replacement	86-year-old man, history of HIT type II	>300	Bolus: 6 mg Infusion: 6mcg/kg/min	>300	None reported	Naganuma et al. 2016 (31)
Mitral valve replacement	63-year-old woman, HIT type II	>500	Infusion: 2.5–65 mcg/kg/min	>500	Prolonged ACT for several hours, 770 cc EBL postoperatively, oxygenator clotting	Follis et al. 2010 (32)
Percutaneous coronary intervention	105 patients received argatroban, RCT compared to heparin	>250	Bolus range: 250, 300, or 350 mcg/kg Infusion: 15, 20, or 25 mcg/kg/min	Few patients in the 250 and 300 groups required additional bolus, and few patients in the 300 and 350 groups required reduced infusion rate	Minor bleeding in 1 patient of the 350 groups	Rossig et al. 2011 (33)

There are few, if any, documented cases in which argatroban is administered for procedural anticoagulation during LAAO. In this study, we describe the case of a patient who received argatroban for procedural anticoagulation during LAAO under general anesthesia due to his history of heparin-induced thrombocytopenia and end-stage renal disease.

## Case presentation

A 67-year-old man (height, 180 cm; weight, 86.5 kg) with a past medical history of HIT type II, heart failure with reduced ejection fraction (LVEF = 35%), atrial fibrillation, coronary artery disease, and end-stage renal disease (ESRD) on intermittent hemodialysis presented for LAAO with the Watchman device. Six months earlier, he had a non-ST segment elevation myocardial infarction believed to be caused by demand ischemia secondary to retroperitoneal and gastrointestinal blood loss while taking apixaban 2.5 mg twice daily. At that time, EGD identified severe gastritis, duodenitis, and multiple upper gastrointestinal ulcers; his apixaban was subsequently discontinued, prompting the need for LAAO. Furthermore, the patient had two documented cases of HIT, 16 and 8 years prior, in which his platelet count reached a nadir of 149,000 cells/mcL and 91,000 cells/mcL, respectively. On both occasions, he documented positive heparin-induced platelet antibody testing and elevated HIT optical density. During the months leading up to his LAAO, his baseline platelet count was consistently below 160,000 cells/mcL, and on the procedure day, his platelet count was 85,000 cells/mcL. His liver studies were within normal limits. After a multidisciplinary discussion, argatroban was selected for intraoperative anticoagulation due to the patient’s history of HIT and his renal dysfunction. Because established dosing of argatroban for LAAO is not distinctly specified in the manufacturer’s labeling, the team utilized recommendations for percutaneous coronary intervention (9). Prior literature (10) calls for a targeted activated clotting time (ACT) of 300 seconds that is achieved via an initial bolus of 350 mcg/kg delivered over 3–5 min followed by an infusion at 25 mcg/kg/min (3).

In the cardiac catheterization lab, general anesthesia was induced intravenously with 100 mcg of fentanyl, 150 mg of propofol, and 50 mg of rocuronium. Intubation was performed with an 8-mm endotracheal tube by direct laryngoscopy using a Macintosh 3 blade. The mean arterial pressure was maintained above 65 mmHg with a norepinephrine infusion running within a range of 2–4 mcg/min. Anticoagulation was monitored using ACT with a goal at or above 300 s as recommended by the drug monograph, which also corroborates with procedural standards (9). Upon case initiation, the baseline ACT was recorded as 136 s.

**Figure 1:**
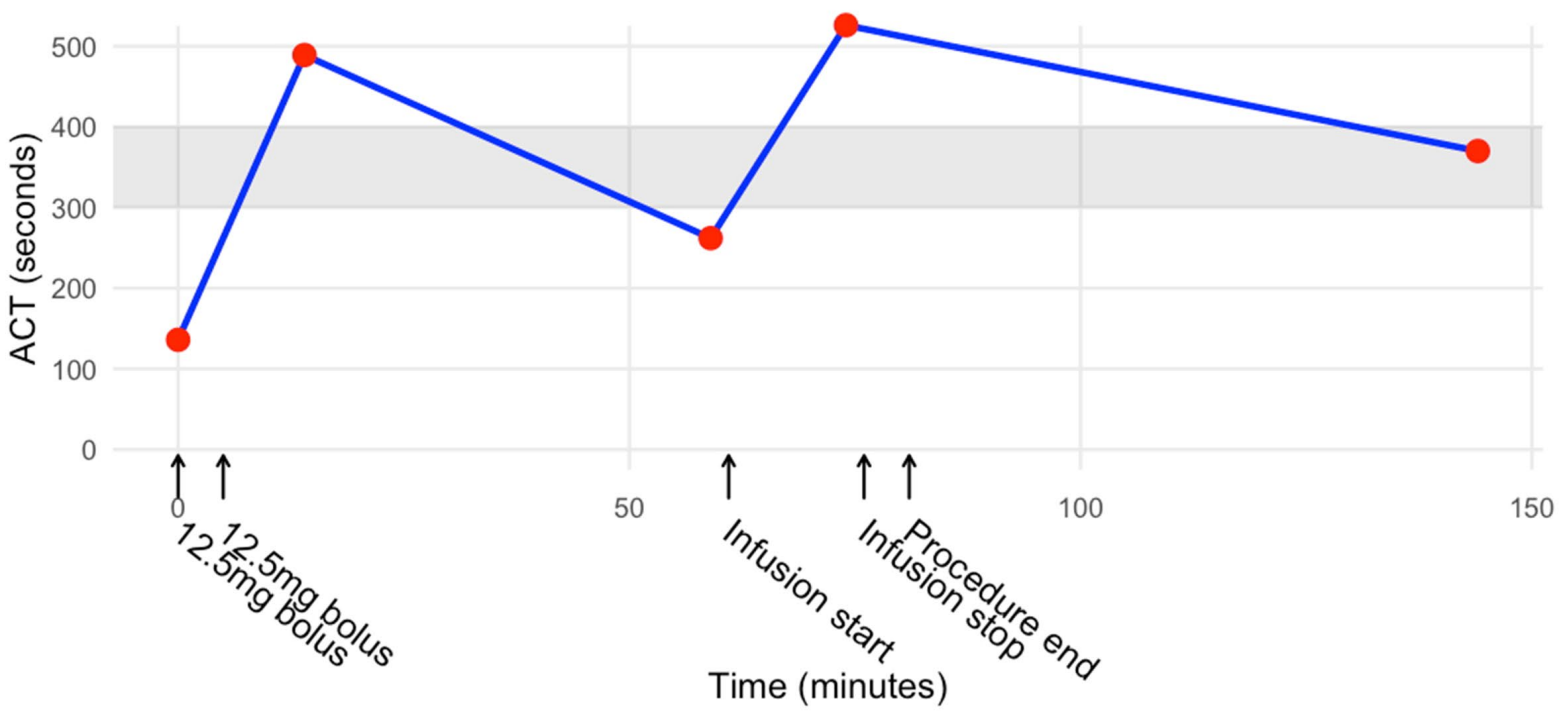
Activated clotting time during and after the procedure. The shaded region from 300 to 400 s indicates the goal range.

Given the concerns about prolonged anticoagulation in this patient, we prepared a reduced dose of argatroban at 290 mcg/kg (25 mg) divided into two boluses of 12.5 mg each for initial anticoagulation. Within 4 min of the first 12.5 mg bolus, the full dose was administered. Five minutes after the administration of the full 25 mg dose, the ACT from a femoral vein sample in the sterile field was 489 s (Figure 1). Because this value was supratherapeutic, the infusion was not initiated. Approximately 45 min after the initial bolus, another sample resulted in an ACT of 262 s, prompting the start of an argatroban infusion at 25 mcg/kg/min. Approximately 5 min after starting the infusion, an additional sample was drawn and resulted in an ACT of 526 s, at which the infusion was stopped. Shortly after, the procedure was completed, and the patient emerged from anesthesia without any complications during extubation or during his stay in the post-anesthesia care unit. The procedure length was 1 h, 35 min. Approximately 1 h after the argatroban infusion was stopped, the patient’s prothrombin time (PT) was 23.0 s, the International Normalized Ratio (INR) was 2.0, and the ACT was 370 s. In accordance with the preoperative plan, the patient was admitted to the hospital to receive hemodialysis the next day. There were no documented intraoperative or postoperative thromboembolic or hemorrhagic complications.

## Discussion

This case highlights the effective use of argatroban for procedural anticoagulation during left atrial appendage occlusion despite a supratherapeutic response, addressing a critical gap in the literature for patients with heparin contraindications. As data on alternative anticoagulants in LAAO remain limited, this report offers practical guidance for clinicians to manage anticoagulation in patients with contraindications to heparin, such as HIT.

Heparin-induced thrombocytopenia type II is caused by immune activation in which antibodies bind to the heparin–platelet factor four complex, resulting in platelet activation, hypercoagulability, and thrombocytopenia (11). Major adverse outcomes in HIT include arterial and venous thrombosis, disseminated intravascular coagulation, and myocardial or cerebral infarction (12). This condition most often occurs in patients who receive heparin for 5 or more consecutive days. HIT type I is a non-immune reduction in platelets that resolves spontaneously, does not increase the risk of thrombosis, and does not require the cessation of heparin (13). Suspicion of HIT type II is driven by clinical indications commonly referred to as the 4 T’s: thrombocytopenia, timing between 5 and 10 days after heparin administration, thrombosis, and exclusion of other causes of thrombocytopenia. ELISA immunoassay is commonly used to assess antibodies to the heparin-platelet factor 4 (HPF4) complex, with optical density indicating the degree of reactivity (13). Confirmation of diagnosis is made with functional testing that measures activation of the HPF4 complex, such as the heparin-induced platelet activation assay and the serotonin release assay, though functional testing is not readily available at many centers (13). Acute management of HIT type II includes terminating all heparin exposure and administering an alternative anticoagulant, typically either a direct thrombin inhibitor or a factor Xa inhibitor.

Heparin remains the drug of choice for procedural anticoagulation due to its ease of administration, established monitoring parameters, the availability of a reversal agent, and low cost (14). Contraindications to using heparin perioperatively include a history of type II HIT, hypersensitivity, active bleeding, or a platelet count below 50,000 cells/mcL. In some cases, a patient can be tested for heparin-PF4 antibodies preoperatively, and if undetectable, heparin can be used intraoperatively if an alternative is not available (15, 16). In our case, the patient had elevated risk for HIT due to his prior documented episodes; additionally, his baseline low platelet count posed a concern for serious bleeding events if another episode of HIT developed.

For individuals who cannot tolerate heparin or who have documented contraindications, alternative anticoagulation regimens can be explored with selection tailored to the unique comorbidities and past medical history of the patient. Generally, organ dysfunction is a major criterion that can make certain medications preferable to others. Both argatroban and bivalirudin are fast-acting intravenous direct thrombin inhibitors with titratable dosing based on coagulation studies; however, their main distinction is adjustments for hepatic or renal disease, respectively (Table 2) (17). Argatroban is safe and effective with varying levels of renal impairment, including patients who receive dialysis therapy (4, 18), though it should be noted that, with hepatic dysfunction, dosages should be reduced due to increased risks of bleeding from reduced metabolism (5). Bivalirudin is essentially the opposite of dosage adjustments: given its high proportion of clearance through the kidneys, it must be used cautiously with renal impairment, particularly in the dialysis population (19, 20).

**Table 2:** Comparison of intravenous anticoagulant medications commonly utilized in cardiovascular procedures.

	Heparin	Bivalirudin	Argatroban
Mechanism	Indirect thrombin inhibitor via antithrombin III	Direct thrombin inhibitor (hirudin analog)	Direct thrombin inhibitor (L-arginine derivative)
Key actions	Inactivates thrombin and factors IXa, Xa, XIa, XIIa, and plasmin	Inhibits thrombin (free and clot-bound), factors V, VIII, and XIII	Inhibits thrombin, factors V, VIII, XIII, and protein C; prevents platelet aggregation
PCI dosing	70–100 units/kg bolus (max 10,000 units) for target ACT 250–300 s	0.75 mg/kg bolus → 1.75 mg/kg/h infusion for target ACT 300–350 s	350 mcg/kg bolus → 25 mcg/kg/min infusion for target ACT 300–450 s
Dose adjustments	None for renal/hepatic	↓ dose if CrCl <30 or if on HD	↓ dose in hepatic impairment
Metabolism and elimination	Hepatic reticuloendothelial system	Hepatic proteolytic cleavage then renally eliminated	Hepatic metabolism (CYP + non-CYP)
Contraindications	HIT, bleeding, hypersensitivity	Bleeding, hypersensitivity	Bleeding, hypersensitivity
Monitoring considerations	Non-linear kinetics; affected by antithrombin 111 levels	Linear prolongation of ACT, aPTT, PT/INR	Linear prolongation of ACT, aPTT, PT/INR
Clinical pearls	Low cost, widely used; reversal with protamine sulfate	Must dilute from powder; renal disease prolongs t½; no reversal agent	Available premixed; increased t½ with hepatic dysfunction; no reversal agent
Approximate cost	$0.40 per 10 mL vial (1,000 U/mL)	$528 per 250 mg/vial lyophilized powder or $1.72 per 5 mg/mL 50 mL vial	$155 per 2.5 mL vial (100 mg/mL) or $4.75 per 50 mL vial (1 mg/mL)

Clinical information obtained from medication package inserts (3, 8, 9). Drug pricing obtained from Merative Micromedex® RED BOOK® (34–36). ACT, activated clotting time; aPTT, activated partial thromboplastin time; CrCl, creatinine clearance; CYP: cytochrome P450 enzyme superfamily; HD, hemodialysis; HIT, heparin-induced thrombocytopenia; INR, international normalized ratio; PCI, percutaneous coronary intervention; PT, prothrombin time; t½, half-life.

The challenge presented by our patient’s case was his clinical picture of HIT combined with ESRD. In particular, the interventional cardiologist had concerns about the consequences of prolonged anticoagulation at the puncture site. After a joint conversation between the attending anesthesiologist, interventional cardiologist, and an inpatient clinical pharmacist, argatroban was selected as the procedural anticoagulant because it did not require dosage adjustments for the patient’s ESRD and would not have a prolonged half-life due to organ failure, unlike bivalirudin. Although the anesthesiology and cardiology teams were more familiar with bivalirudin dosing and use for other catheter-based cardiac procedures, argatroban was safely and effectively utilized in this patient, further demonstrating its use as a viable alternative to heparin.

While the use of bivalirudin and argatroban is well-documented in the literature for the treatment of HIT (4, 5, 16–21), sparse case reports exist on their usage in cardiac surgery or catheterization-based procedures. Regarding the use of argatroban as a procedural anticoagulant during LAAO with a Watchman device, we were unable to find any documented reports in the medical literature. Likely because of cardiologists’ familiarity with bivalirudin, this medication is often selected over argatroban in patients with a history of HIT (22, 23). In this case, we demonstrate that argatroban can be safely used for intraoperative anticoagulation in patients with contraindications to heparin and bivalirudin. Despite using a 17% lower initial bolus than recommended, this patient was quickly above the goal ACT and did not require an immediate infusion. When the infusion was started at the recommended dose, the patient again rapidly became supratherapeutic. This supratherapeutic response to a standard or lower dosing of argatroban may indicate that caution should be exercised in these patients and emphasizes the importance of frequent ACT monitoring. In addition to argatroban, other unmeasured confounders may contribute to prolonged ACT, such as uremic platelet dysfunction or platelet factor deficiencies. It is particularly concerning when there is no specific reversal agent and patients may remain significantly anticoagulated postoperatively, as was observed with this patient. i-STAT ACT (iACT) monitoring was originally approved for use during procedures with heparin anticoagulation (24). It has been reported that i-STAT underestimates the ACT compared to Hemochron (hACT) for therapeutic levels of heparin anticoagulation (25). An additional study found that iACT readings were consistently lower than hACT following heparin administration, that both measures showed poor correlation with anti-Xa levels, and that post-heparin iACTs that met procedural thresholds were associated with supratherapeutic TEG R-times, suggesting that, even within accepted ACT ranges, supratherapeutic anticoagulation may already be present (26). The use of iACT has also been validated compared to hACT during bivalirudin administration (27), yet there are no published studies validating the use of iACT in argatroban, further adding to the uncertainty in the use of argatroban for procedural anticoagulation. Although i-STAT ACT lacks validation for argatroban, we relied upon it because it was the fastest and most convenient point of care testing for intraoperative titration of argatroban during our case. Extensive anticoagulation is particularly dangerous in patients undergoing LAAO, as the indication for the procedure is often intolerance to oral anticoagulation secondary to hemorrhage. Our patient had a mild decrease from preoperative to postoperative day 1 in hemoglobin (−0.7 g/dL) but this finding could be accounted for by the standard error of lab measurements given there were no major hemorrhagic complications or a puncture site hematoma.

Our experience with argatroban better informs future intraoperative use. Recognizing that standard dosing guidelines can lead to anticoagulation beyond the target range underscores the importance of closely monitoring coagulation studies. We utilized ACT, as it can be rapidly assessed in the operating room. We did not measure an ACT after the first half-dose of the initial bolus; doing so could have influenced our management if the results approached therapeutic levels and might have prevented excessive anticoagulation. An even greater reduction in the initial bolus dose and a lower infusion rate could have been ideal for the target ACT of 300. Further investigation is warranted to establish clear argatroban dosing guidelines in LAAO; multicenter trials in addition to i-STAT ACT validation for argatroban would better inform clinical management.

## Conclusion

Argatroban can be safely administered as an intraoperative anticoagulation agent in a patient with renal failure and a history of HIT undergoing structural cardiac intervention. The use of a lower than recommended initial dose may be warranted to avoid excessive anticoagulation. Proper dosing is patient-dependent and requires careful titration to achieve the goal of anticoagulation.

## Data Availability

The original contributions presented in the study are included in the article/supplementary material, further inquiries can be directed to the corresponding author.
